# Changes in use of herbs and dietary supplements (HDS) among clinicians enrolled in an online curriculum

**DOI:** 10.1186/1472-6882-7-21

**Published:** 2007-06-12

**Authors:** Kathi J Kemper, Paula Gardiner, Charles Woods

**Affiliations:** 1 Department of Pediatrics, Wake Forest University School of Medicine, USA; 2 Division for Research and Education in Complementary and Integrative Medical Therapies, Harvard Medical School, Boston, Massachusetts; Division of General Medicine and Primary Care, Beth Israel Deaconess Medical Center, Boston, MA, USA; 3 Department of Pediatrics, University of Louisville School of Medicine, Louisville, KY, USA

## Abstract

**Background:**

Little is known about clinicians' use of herbs and dietary supplements (HDS), how their personal HDS use changes with time and training, and how changes in their personal use affect their confidence or communication with patients about HDS.

**Methods:**

We conducted a prospective cohort study of clinicians before and after an on-line curriculum about HDS in winter-spring, 2005.

**Results:**

Of the 569 clinicians who completed surveys both at baseline and after the course, 25% were male and the average age was 42 years old; 88% used HDS before and after the course. The average number of supplements used fell slightly from 6.2 at baseline to 5.8 after the course (P < 0.01). The most commonly used supplements at baseline were: multivitamins (65%), calcium (42%), B vitamins (34%), vitamin C (34%), green tea (27%), fish oil (27%) and vitamin E (25%). Use of fish oil increased to 30% after the course (P = 0.01). Use of supplements traditionally used to treat colds decreased: vitamin C (34% to 27%), zinc (13% to 10%), and echinacea (7% to 5%, P < 0.05 for all three). Changes in personal HDS use were not associated with significant changes in confidence or communication with patients.

**Conclusion:**

Many clinicians use HDS personally; use changes seasonally and to a small extent with professional education. Professional use of HDS is dynamic and seasonal. Additional research is needed to understand the impact of personal use on professional attitudes and behavior in populations with lower baseline uses of HDS.

## Background

Herbs and other dietary supplements (HDS) such as vitamins and minerals are among the most commonly used complementary therapies in the US [1-5]. Substantial numbers of all patient groups report using HDS, particularly those with chronic or recurrent illnesses. Rates of use have been shown to vary by demographic characteristics. For example, compared with men, women report higher use of HDS [6-9]. Among health care professionals (clinicians), HDS use tends to mirror patterns reported by the general public [10-16].

Clinicians' personal use of dietary supplements is of interest for at least two reasons[15]. Patients ask clinicians to provide advice about them. In fact, the labels on dietary supplement products frequently suggest that consumers ask clinicians for advice about using such products. Furthermore, clinicians' personal health habits may affect their professional behavior[3].

We previously reported a study comparing four different strategies for delivering on-line curriculum about HDS. All four delivery strategies were associated with similar, significant and sustained improvements in participants' knowledge, confidence and communication practices both immediately after and six to ten months after completing the course[17]. The on-line course included 40 self-instructional modules covering scientific evidence of risks, safety, and effectiveness of over 100 commonly used HDS; the modules were organized into ten topic areas covering common clinical questions and situations such as safety, women's health, aging, pediatrics, cardiology, pulmonary and gastrointestinal problems. Each module included links to additional sources of evidence-based clinical information.

We wondered whether completing the on-line curriculum (geared toward helping clinicians learn to answer patients' questions about HDS) might also affect clinicians' personal use of HDS because clinicians may also be interested in improving their own health or addressing their own health problems as well as those of patients. We expected that use of some HDS would be relatively stable over time (e.g. use of multivitamins, calcium and folate), while others would change seasonally (e.g., use of vitamin C, echinacea and zinc used to prevent or treat the common cold would decrease as cold/flu season ended) and some might change as a result of information provided during the course. Furthermore, we were curious about the relationship between changes in personal use of HDS and changes in communication practices.

## Methods

To answer these questions, we prospectively compared participants' baseline (January) surveys with their responses to follow-up surveys completed within a month after course completion in spring (April – May), 2005. Subjects were eligible if they were participants enrolled in a randomized controlled trial evaluating different strategies for delivering an on-line curriculum about HDS. The eligibility criteria, recruitment strategies, curriculum content, and delivery strategies have been described previously [17-19] as have analyses of participants' personal use of HDS at baseline [16].

Briefly, participants were eligible if they were physicians, nurses, dietitians, pharmacists or trainees in one of these professions. They were recruited through email, a web site, flyers and mailings from the North Carolina Northwest Area Health Education Center (NW AHEC). Enrollment and follow-up surveys were completed on-line.

The surveys were based on our earlier pilot study evaluating the effects of an email-based curriculum about HDS [20]. They included questions about demographic and practice characteristics, personal use in the previous week of a list of 15 vitamins and minerals, 59 herbs and 12 other dietary supplements. The survey also included questions to assess participants' knowledge, confidence and communication practices about commonly used HDS [18] [see Additional file 1]; the confidence and communication scales had Cronbach alpha > 0.7. The same questions were used for both the baseline and follow-up surveys.

This analysis was restricted to participants from the winter-spring 2005 semester to assess seasonal impact on HDS use. For purposes of this study, we defined as "seasonal supplements": vitamin C, echinacea, zinc, astragalus, slippery elm bark, chamomile, elderberry, ginger, goldenseal, licorice, medicinal mushrooms and peppermint.

Analysis included simple descriptive statistics such as mean and standard deviation for normally distributed data; and medians and ranges for non-normally distributed data. We used Chi square methods to assess associations for categorical variables. Spearman correlation was used to assess associations for continuous variables. Mann-Whitney and Kruskal-Wallis tests were used to assess between group differences. Wilcoxon signed rank and McNemar tests were used to assess repeated measures for continuous and binomial measures, respectively.

All analyses were performed using SPSS 14.0 (SPSS, Inc., Chicago, IL).

This study was approved as "exempt" as an educational research project by the Wake Forest University School of Medicine Institutional Review Board.

## Results

Of the 1268 course participants, 569 were spring enrollees who completed baseline and follow-up questionnaires (Table 1). At baseline the participants' average age was 40.3 ± 12.9 years and 25% percent were men. At baseline, 88% of enrollees used at least one dietary supplement and the average number used was 6.2. Table 2 lists the most commonly used HDS which included: multivitamins, calcium, B vitamins, vitamin C, green tea, and fish oil. Higher baseline use of HDS was associated with older age, female gender, professional group (highest in nurses and lowest in students and pharmacists), and higher scores on knowledge questions, the confidence scale and the communication practices scale [15].

**Table 1:** Characteristics of and dietary supplement use among 569 health professionals at baseline (winter) and following course (spring)

*Characteristic*	N (%)*	*No. Products Used at Baseline (median)*	*P*	*Change in No. Products Used at Baseline (median)*	*P*
Age, yrs (mean ± SD)	41.8 ± 12.9	6.2 ± 6.2	--	-0.44 ± 4.2	.006‡
Age			< .001§		.09§
Age < 30 yrs	163 (29)	4.5 ± 5.1 (3)		-0.94 ± 4.0 (0)	
Age 31–40 yrs	78 (14)	5.0 ± 5.0 (3)		-0.04 ± 5.2 (0)	
Age 41–50 yrs	167 (29)	7.1 ± 7.0 (5)		-0.44 ± 4.0 (0)	
Age > 50 yrs	161 (28)	7.4 ± 6.3 (6)		-0.14 ± 3.9 (0)	
					
Gender			.003†		.70†
Male	141 (25)	5.1 ± 5.7 (3)		-0.31 ± 3.6 (0)	
Female	428 (75)	6.5 ± 6.3 (5)		-0.49 ± 4.3 (0)	
Race/Ethnicity			.27†		.39†
Caucasian	480 (84)	6.3 ± 6.3 (4)		-0.40 ± 4.3 (0)	
Other	89 (16)	5.4 ± 5.2 (5)		-0.69 ± 3.5 (0)	
Profession			< .001§		.30§
Physician/PA	170 (30)	6.2 ± 5.7 (5)		-0.31 ± 4.0 (0)	
Dietician	65 (11)	4.7 ± 5.4 (3)		-0.72 ± 4.2 (0)	
Nurse	153 (27)	8.0 ± 7.0 (6)		-0.18 ± 3.8 (0)	
Pharmacist	19 (3)	5.3 ± 5.6 (3)		+0.58 ± 7.0 (0)	
Student	162 (28)	5.0 ± 5.7 (3)		-0.84 ± 4.2 (-.5)	
					
Practice status			< .001†		.024†
Faculty/In practice	349 (61)	6.9 ± 6.5 (5)		-0.26 ± 4.2 (0)	
Trainee	220 (39)	4.9 ± 5.4 (3)		-0.75 ± 4.0 (0)	
					
Saw Patients in 30 days prior to questionnaire			.14†		.12†
Yes	425 (75)	6.3 ± 6.0 (5)		-0.36 ± 4.2 (0)	
No	144 (25)	5.8 ± 6.5 (3)		-0.69 ± 4.0 (0)	
					
Knowledge Scores (%Correct)	N = 561^A^	Correlation coefficient**		Correlation coefficient**	
Baseline	66.2 ± 10.3	.30	< .001	-.03	.45
Outcome	90.0 ± 8.7	.03	.51	.04	.39
Change	23.8 ± 12.5	-.20	< .001	.03	.47
Confidence Scale Score (possible range, 19–95)	N = 525^B^				
Baseline	49.0 ± 16.7	.38	< .001	-.06	.16
Outcome	70.9 ± 10.5	.25	< .001	.02	.58
Change	21.9 ± 14.1	-.27	< .001	.07	.13
Communications Practices Scale Score (possible range, 0–10)	N = 386^C^				
Baseline	2.43 ± 2.05	.26	< .001	-.03	.52
Outcome	2.67 ± 2.30	.22	< .001	-.01	.82
Change	0.24 ± 1.79	.08	.14	-.04	.40

^A ^For knowledge assessments, 561 of the enrollees completed baseline and outcomes assessments. P < .001 for change (Wilcoxon signed ranks test). Mean age was 41.8 yrs.

^B ^For confidence assessments, 525 of the enrollees completed baseline and outcomes assessments. P < .001 for change (Wilcoxon signed ranks test). Mean age was 41.7 yrs.

^C ^For communications practices, 386 of the enrollees completed baseline and outcomes assessments. Enrollees had to have seen patients in the 30 days prior to complete this section of the assessment. P = .005 for change (Wilcoxon signed ranks test). Mean age was 43.9 yrs.

*Unless specified otherwise.

† Mann-Whitney test.

‡ Wilcoxon signed rank test.

§Kruskal-Wallis test.

** Spearman rank correlation coefficient.

**Table 2:** Changes in the use among the most frequently used HDS

Herb or Dietary Supplement	Baseline		After Course		P value*
	N	%	N	%	
Multivitamins	367	64.5	362	63.6	0.743
Calcium	237	41.7	242	42.5	0.742
B vitamins	193	33.9	174	30.6	0.081
**Vitamin C**	**191**	**33.6**	**152**	**26.7**	**0.001**
Green tea	154	27.1	143	25.1	0.359
Fish Oil	151	26.5	171	30.1	0.035
Vitamin E	143	25.1	111	19.5	0.002
Vitamin D	100	17.6	103	18.1	0.838
Flax seed	99	17.4	100	17.6	0.999
Folate	89	15.6	86	15.1	0.828
Magnesium	85	14.9	77	13.5	0.374
Glucosamine	78	13.7	77	13.5	0.999
**Zinc**	**76**	**13.4**	**57**	**10**	**0.023**
Vitamin A	74	13	59	10.4	0.105
Coenzyme Q	68	12	69	12.1	0.999
Probiotics	63	11.1	56	9.8	0.371
**Chamomile**	**59**	**10.4**	**49**	**8.6**	**0.193**
Iron	58	10.2	36	6.3	0.005
Cinnamon	49	8.6	44	7.7	0.568
Niacin	45	7.9	47	8.3	0.878
Aloe vera	42	7.4	29	5.1	0.098
**Echinacea**	**41**	**7.2**	**27**	**4.7**	**0.034**
**Peppermint**	**39**	**6.9**	**37**	**6.5**	**0.875**
Vitamin K	38	6.7	28	4.9	0.164
Chondroitin	37	6.5	32	5.6	0.424
Alpha lipoic acid	36	6.3	35	6.2	0.999
Selenium	34	6	40	7	0.392
Teatree oil	34	6	41	7.2	0.349
Garlic	33	5.8	28	4.9	0.442
MSM	30	5.3	29	5.1	0.999
Lavender	29	5.1	28	4.9	0.999

* McNemar test

NOTE: Bold items were pre-specified as being seasonal HDS products.

Overall, there was little change in the use of HDS from before to after the course. After the course, 88% of enrollees still reported using at least one dietary supplement; the average number of supplements used fell slightly from 6.2 to 5.8 (P < 0.01). As expected, there were significant decreases in the use of seasonal HDS (Table 3). For example, use of vitamin C fell from 34% to 27% (P = 0.001). Overall, the average number of seasonal HDS products used fell more than 20% from baseline to after the course (P < 0.001), while the average number of non-seasonal products used fell less than 5% (P = 0.08). There were unexpectedly significant changes in the use of three non-seasonal HDS: an increase in the use of fish oil from 27% to 30% (P = 0.04) and declines in the use of vitamin E (25% to 20%, P = 0.002) and iron (10% to 6%, P = 0.005). Exploratory statistical analyses did not reveal any pattern of associations between use of fish oil, iron or vitamin E and demographic or practice characteristics.

**Table 3:** Changes in Seasonal versus non-Seasonal HDS

**Category**	**Baseline Use (mean ± SD)**	**Change in Use (mean ± SD)**	**P**
Total HDS Use	6.2 ± 6.2	-0.44 ± 4.2	**.006**
Seasonal Products	0.87 ± 1.2	-0.19 ± 1.0	**< .001**
Non-seasonal Products	5.3 ± 5.3	-0.25 ± 3.6	**.083**

Seasonal products include: astragalus, chamomile, echinacea, elderberry, ginger, goldenseal, licorice, medicinal mushrooms, peppermint vitamin C, slippery elm bark, zinc

Higher use of HDS at baseline was associated with higher scores on knowledge, confidence and communication practices scales and significantly greater improvements in knowledge and confidence after the course. However, there were no significant associations between *changes *in HDS use and improvements in knowledge, confidence or communication practices from before to after the course. Trainees reported a greater decrease in use of HDS than did practitioners (P = 0.02), but there were no significant associations between changes in HDS use and other baseline characteristics.

## Discussion

This is the first study to examine changes in professionals' use of HDS over time, the impact of these changes associated with changes in professional behavior, and the impact of an on-line professional curriculum and seasonal changes on clinicians' personal use of these products. Generally, we found little change in HDS use over time except for the expected decrease in the use of seasonal cold/flu products. Unexpected changes included a significant increase in the use of fish oil (which was discussed in several modules as potentially helpful) and decreases in the use of iron and vitamin E (which were mentioned in modules, but not heavily emphasized).

Overall, the clinicians in this study used more HDS than the general public [1,2,21]. Although the self-selected participants in this study had higher usage rates, their patterns of use, i.e., the most popular supplements, were generally similar to those used by the general public. For example, in both these participants and the general public, multivitamins are the most commonly used supplements. On the other hand, our participants reported lower usage of supplements traditionally used to treat colds and flu (e.g. vitamin C, echinacea and zinc) than the general public. The lower use in our study may be due to the healthy worker effect [22]; on the other hand, we did not collect personal health information, and it is possible that the participants in this study had higher rates of chronic conditions that led to their greater use of HDS than that reported among the general public. Perhaps due to recent positive trials about the benefits of fish oil for a variety of health conditions, reported use of fish oil among our participants was higher than that previously reported among the general public[1,2,21]. Likewise, the decreasing use of vitamin E over time among our enrollees may have been due to course coverage of recent randomized trials casting doubt on its effectiveness in preventing heart disease as had been claimed in earlier epidemiologic studies.

This study has several limitations. First, we enrolled a highly self-selected sample of clinicians who were very high users of HDS before starting the course; results from this sample may not be generalizable to other groups of clinicians. Second, we did not collect personal health data or reasons for using HDS; this makes it difficult to speculate on reasons for changes in use beyond the obvious seasonal changes. Furthermore, we had no control over secular changes in information about HDS which might have influenced personal use; for example, during the study period, participants may have been exposed to articles in professional journals or the popular media about vitamin E, fish oil and seasonal remedies such as echinacea. Also, the curriculum provided information on more than 100 HDS; individual participants may have increased or decreased their personal use of individual supplements in significant ways not captured by the aggregate data. Given these limitations, we cannot speculate on the effect of the content of the curriculum on individual clinician's use of specific supplements. For example, we cannot tell whether increases in the use of fish oil or decreases in the use of vitamin E and iron are related to curriculum content, personal health factors, secular changes in supplement use, or simply artifacts related to testing changes in multiple supplements.

## Conclusion

Although higher use of HDS is associated with greater knowledge and confidence in talking with patients about HDS, small seasonal changes in personal use of HDS do not appear to affect clinical behavior. Use of HDS is dynamic; use of some supplements varies seasonally and may be affected by numerous factors. Prevalence studies alone are insufficient to understand important facets of HDS use. Prospective cohort studies that include data on health conditions and use of over the counter and prescription medications are needed to better understand the scope and effects of HDS use among clinicians as well as patients.

## Competing interests

The author(s) declare that they have no competing interests.

## Authors' contributions

KK conceived the project, wrote the initial drafts of the manuscript and revised the manuscript

PG assisted in developing the idea of the project, developing the study intervention, defined key parameters and assisted in drafting and revising the manuscript

CW assisted in designing the project, analyzing study data and revising the manuscript

## Pre-publication history

The pre-publication history for this paper can be accessed here:


